# Lysophosphatidic acid-RAGE axis promotes lung and mammary oncogenesis via protein kinase B and regulating tumor microenvironment

**DOI:** 10.1186/s12964-020-00666-y

**Published:** 2020-10-27

**Authors:** Rashmi Ray, Nitish Jangde, Satyendra Kumar Singh, Sunita Sinha, Vivek Rai

**Affiliations:** 1 Laboratory of Vascular Immunology, Institute of Life Sciences, (An Autonomous Institute of Department of Biotechnology (DBT) New Delhi), Bhubaneswar, 751023 India; 2 Manipal Academy of Higher Education, Manipal, Karnataka-576104 India

**Keywords:** Cancer, Tumorigenesis, Metastasis, Migration, Invasion

## Abstract

**Background:**

Receptor for advanced glycation end products (RAGE) is a multi-ligand transmembrane receptor of the immunoglobulin superfamily. Lysophosphatidic acid (LPA) is a ligand for RAGE and is involved in physiological and pathophysiological conditions including cancer. However, RAGE-LPA axis is unexplored in lung and mammary cancer.

**Methods:**

RAGE was silenced in A549, MDA MB-231 and MCF7 using RAGE shRNA. For in vitro tumorigenesis, we performed wound healing, colony formation, cell proliferation and invasion assays. Evaluation of expression of oncogenes, EMT markers and downstream signaling molecules was done by using western blot and immunohistochemistry. For subcellular expression of RAGE, immunofluorescence was done. In vivo tumorigenesis was assessed by intraperitoneal injection of cancer cells in nude mice.

**Results:**

Here we show RAGE mediated profound increase in proliferation, migration and invasion of lung and mammary cancer cells via LPA in Protein kinase B (PKB) dependent manner. LPA mediated EMT transition is regulated by RAGE. In vivo xenograft results show significance of RAGE in LPA mediated lung and mammary tumor progression, angiogenesis and immune cell infiltration to tumor microenvironment.

**Conclusion:**

Our results establish the significance and involvement of RAGE in LPA mediated lung and mammary tumor progression and EMT transition via RAGE. RAGE-LPA axis may be a therapeutic target in lung and mammary cancer treatment strategies.

Video Abstract

**Supplementary information:**

**Supplementary information** accompanies this paper at 10.1186/s12964-020-00666-y.

## Background

Cancer accounts for the major global health problem with lung and mammary cancer being the most frequent among all. Lung cancer covers a major percentage of cancer-related mortality with non-small-cell lung cancer (NSCLC; 85 to 90% of all lung cancers) being the most common form [1]. Patients with aggressive lung tumors show very poor survival rate, due to its metastasis. Breast cancer ranks second after lung cancer and develops in ducts and lobules. Lung and breast cancer progression is a complex biological phenomenon and in spite of several decades of research, the detailed molecular mechanisms still remain elusive.

Receptor for advanced glycation end products (RAGE) is a multi-ligand transmembrane receptor belonging to immunoglobulin superfamily. RAGE is expressed on different cell types specifically- endothelial cells, smooth muscle cells, cardiac myocytes, immune cells and neural tissue [2]. RAGE is upregulated in inflammatory and pathophysiological conditions and is associated with diseases such as diabetes, vascular dysfunction, neurodegenerative disorders, Alzheimer’s disease [3–10]. RAGE structure consists of three domains viz. an extracellular domain with V, C1 and C2, a transmembrane domain and a short cytoplasmic tail [11, 12]. RAGE extracellular domain binds to various ligands including advanced glycation end products (AGEs), amyloid beta (aβ), S100B proteins/calgranulins, high mobility group box proteins (HMGB1), phosphatidylserine and lysophosphatidic acid (LPA) [13–16]. RAGE is found to be associated with tumor progression in glioma, bladder, melanoma, liver, pancreatic, prostate, colorectal, ovarian, gastric and lung cancer [15, 17–19]. RAGE-ligand interaction leads to activation of distinct signaling pathways - Rac-1, MAP kinase family (ERK, p38 and SAPK/JNK) and NF-κB resulting in the regulation of cellular migration and invasion. Furthermore, RAGE is also shown to be involved in epithelial to mesenchymal transition in mammary tumor microenvironment [20]. Blocking RAGE signaling inhibits cancer cell growth in vitro and reduce tumorigenicity in murine models [21, 22].

Lysophosphatidic acid (LPA) is a biologically active phospholipid present in plasma, tissues and is shown to be involved in normal and pathophysiological conditions such as atherosclerosis, inflammation, diabetes and cancer [15, 23]. LPA is produced from lysophosphatidylcholine by the catalytic activity of ectoenzyme autotaxin. LPA binds to many G-protein coupled receptors (GPCRs) viz. LPAR (1–6), GPR87 and GPR35, RAGE, P2Y10 and intracellularly to TRPV1 [24, 25]. LPA receptors show different level of distribution in tissues and differ in downstream signalling. LPA is involved in various cellular processes such as proliferation, migration, differentiation, tissue invasion of immune cells and cancer cells [26–28] and higher levels of LPA are found in inflammation and tumors [29–36]. However, involvement of LPA-RAGE axis in driving tumor development, metastasis and modulation of tumor microenvironment in lung and breast cancer is unknown. Here, we show that LPA induces lung and mammary tumor proliferation, invasion and tumorigenesis via RAGE. Furthermore, our result establishes Protein kinase B (PKB) pathway involvement in LPA-RAGE mediated signalling and EMT transition in these cancers. Targeting LPA-RAGE axis may serve as a therapeutic avenue in these cancers.

## Materials and methods

### Animals

All animal protocols were approved by the Institute of Life Sciences Animal Care and Use Committee. All animals used in the experiments were of BALB/c background athymic (Nude mice) and 6–8 weeks of age.

### Cell lines and culture

We obtained A549, MCF-7 and MDA MB-231 cancer cell line from NCCS, Pune. MDA MB-231 and MCF-7 cells were cultured in DMEM with 10% FBS and A549 cells were maintained in DMEM-F12 medium supplemented with 10% FBS, streptomycin (100 μg/mL) and penicillin (100 μg/mL). Cells were maintained in an incubator with a humidified atmosphere containing 5% CO_2_ at 37 °C.

### LPA treatment

For in vitro studies, 5 μM LPA was used after overnight serum starvation. For in vivo experiments, 20 μg LPA in 100 μl of PBS was injected into the peritoneum of BALB/c nude mice every alternate day for 21 days.

### RAGE Lentiviral transduction

High-titer RAGE lentiviral particles were procured from Sigma (SHCLNV-NM_001136, TRCN0000371281). A549, MDA MB-231 and MCF-7 cells cultured in 96 well plates were infected with control shRNA, RAGE shRNA in opti-MEM with polybrene (8 μg/ml). shRNA infected cells were left overnight at 37 °C and 5% CO_2_. Medium was changed the next day and was replaced with fresh medium for 24 h. After 24 h of resting, RAGE transfected cells colonies were selected from cells grown in medium with 1 μg/ml Puromycin. Puromycin selected colonies were evaluated for RAGE silencing using western blotting and qPCR for RAGE expression.

### PKB inhibitor treatment

Cells were pre-treated with 10 μM of Protein kinase B (PKB) inhibitor (LY294002) for 1 h for cell migration, proliferation and cell invasion assays. After one-hour incubation with PKB inhibitor, cells were stimulated with 5 μM LPA and cultured for indicated time points for cell migration and invasion assays.

### Invasion assay

The effect of LPA on RAGE silenced A549, MDA MB-231 and MCF-7 cells invasion was evaluated by Transwell migration assay. After RAGE shRNA transduction, 5 × 10^3^ A549 cells in 200ul DMEM medium were plated into the upper chamber of the Transwell chamber with collagen coated polycarbonate membrane (8.0 μm, Corning). Five hundred microliter DMEM medium containing 5 μM LPA was added to the Transwell lower chamber. After incubation for 6 h, A549, MDA MB-231 and MCF-7 cells on the lower surface of polycarbonate membrane were fixed with 4% paraformaldehyde and stained with crystal violet. The non-migrated A549, MDA MB-231 and MCF-7 cells on the upper peripheral -side of the polycarbonate membrane were removed with a cotton swab. Image of the A549, MDA MB-231 and MCF-7 cells which migrated to the under-surface of the membrane was captured at 5 different microscopic fields. The number of A549, MDA MB-231 and MCF-7 cells invaded to lower chamber were counted from five randomly fields per polycarbonate membrane. Cell invasion assay was performed in a similar way for PKB inhibitor treated A549, MDA MB-231 and MCF-7 cells where cells were pre-treated with PKB inhibitor and then observed for cell invasion property with LPA stimulation.

### Clonogenic assay

A549, MDA MB-231 and MCF-7 cells clonogenic assay was performed to measure the growth ability of single A549 cell to grow into a colony in vitro. Briefly, after transducing A549, MDA MB-231 and MCF-7 cells with RAGE shRNA and control shRNA, cells were seeded in complete DMEM media at a density of 1 × 10^3^ cells in 6 well plates. The plates were incubated for 2 weeks at 37 °C, followed by fixation with 1% methanol (v/v) and then stained with 0.1% crystal violet. Colonies with greater than 50 cells were counted manually.

### Cell proliferation assay

Cell viability assay was performed to check the proliferation rate of A549, MDA MB-231 and MCF-7 cells. Cells were seeded in 96-well cell culture plates at a density of 1 × 10^6^ cells/well. After incubation for 24 h, 48 h or 72 h, respectively, 100 μl of 3-(4,5-Dimethylthiazol-2-Yl)-2,5-Diphenyltetrazolium Bromide (MTT, Sigma) (5 mg/ml) was added to the cells for 4 h. The farmazan crystals formed after 4 h incubation were dissolved by adding solvation buffer (DMSO: isopropanol; 1:1) for 30 min. The absorbance of each well was acquired at 570 nm with the help of ELISA plate reader (Synergy™ HT Multi-Mode Microplate Reader (Bio-Tek, Winooski, VT, USA).

### Wound healing assays

Cell migration ability of A549, MDA MB-231 and MCF-7 cells on LPA treatment was detected by scratch assay. A549, MDA MB-231 and MCF-7 cells were seeded in 6 well plates at the density of 3 × 10^5^ cells/well. After 24 h when A549, MDA MB-231 and MCF-7 cells reached 90–100% monolayer confluence, they were serum starved for overnight. After overnight serum starvation, a straight scratch was artificially created in the cell monolayers with 200 μl sterile pipette tip. Cells debris by the scratch was removed with phosphate buffer saline (PBS) and cultures were then supplemented with fresh DMEM/F-12 medium alone, DMEM/F-12 with 5% FBS as positive control and DMEM/F-12 with 5 μM LPA for 24 h at 37 °C. Migration images were captured using an inverted microscope (CarlZeiss, Germany). The scratch wound widths were measured under microscope and the relative percentage of wound closure was determined by comparing to control cells. Migration ability of RAGE silenced A549, MDA MB-231 and MCF-7 cells on LPA stimulation was also detected in a similar way after RAGE silencing by shRNA.

### Immunofluorescence

For immunocytochemistry (ICC), control and RAGE shRNA infected A549, MDA MB-231 and MCF-7 cells were grown on coverslips in 6 well plates in DMEM with 10% FBS and 1% Penicillin streptomycin. Cells were given overnight serum starvation followed by treatment with 5 μM LPA for indicated time points. After completion of the indicated time points, cells were fixed in methanol and permeabilised with 0.1% triton-X 100 and blocked with 3% BSA in PBS for 30 min followed by staining with phalloidin for 4 h and counterstaining by DAPI. Cells were mounted in mounting medium and imaged by using Leica confocal imaging software on Leica microscope.

### Immunoblot analysis

Total protein extract was obtained by homogenizing A549, MDA MB-231 and MCF-7 cells in cell lysis buffer (Cell Signaling) added with 1 mM PMSF and 1 mM sodium fluoride (NaF) and incubated at 4 °C for 30 min, with frequent agitation for 1 min at every 5 min interval. Cell debris was removed by centrifugation at 12,000 g for 10 min at 4 degrees, and the supernatant was collected and stored at − 80 °C. Protein concentration was determined using Bradford protein assay method. Total protein extracts (40 μg) were then electrophoresed in 10% acrylamide/bis-acrylamide SDS gels. Gels were transferred to nitrocellulose membranes. Membranes were blocked in 1× Tris-buffered saline (TBS) and 0.1% Tween-20 (TBST) with 5% (w/v) skimmed milk at room temperature for 1 h, followed by an overnight incubation with diluted primary antibody in blocking buffer at 4 °C with gentle shaking. After washing with TBST, the membranes were incubated at room temperature for 1 h with polyclonal anti-rabbit and anti-mouse IgG secondary antibodies conjugated with horseradish peroxidase (HRP) (Amersham, 1:5000 dilution). Membranes were visualized with enhanced chemiluminescence, followed by exposure to film. The primary antibodies used were as follows: RAGE (SC-365154, Santacruz, 1:1000 dilution), p-ERK (9102S)/ERK (9106S, Cell Signaling, 1:1000 dilution), p-PKB (4060S)/PKB (4685S, Cell Signaling, 1:1000 dilution), p-STAT3 (9138S)/STAT3 (12640S, Cell Signaling, 1:1000 dilution), c-myc (9402S, Cell Signaling, 1:1000 dilution), cyclinD1 (SC-753, Santacruz, 1:1000 dilution), snail (3879S, Cell Signaling, 1:1000) slug (9585S, Cell Signaling, 1:1000), Twist (SC-81417, Santa Cruz, 1:1000), and rabbit anti-GAPDH (2118S, Cell signaling).

### Xenograft tumor assay

Female BALB/c nu/nu mice (4–6-weeks-old) were obtained from Advanced Centre for Treatment, Research and Education in Cancer (ACTREC), India and maintained in specific pathogen-free conditions. For the intraperitoneal tumor model, A549, MDA MB-231 and MCF-7 cells transduced with control and RAGE shRNA were suspended with PBS at a concentration of 2.5 × 10^5^ cells/100 μl. A volume of 0.1 ml cells was intraperitoneally injected into mice and mice were observed for 3 weeks. The xenograft tumor number and volume were counted and measured at experimental end point. Tumor specimen were fixed in 10% formalin, embedded in paraffin and sectioned at 5 μm.

### Immunohistochemical analysis

For immunohistochemical assay, endogenous peroxidases were blocked in 3% hydrogen peroxide in methanol. These sections were probed with primary antibody Ki-67 (Ab15580), CD34 (sc-7324), CD45 (M0701, Dako) and F4/80 (sc-26,643). E-cadherin and Vimentin antibodies were also used for IHC analysis. Tumor tissue sections slides were subsequently incubated with HRP tagged secondary antibody. Staining was visualized with 3,3′-diaminobenzidine (DAB) chromogen and counterstained with haematoxylin. The IHC DAB stained images intensity were quantified as percentage area by using ImageJ software.

### Statistical analyses

Data are analysed and presented as mean and std. dev. using Microsoft Excel and Graphpad Prism (GraphPad Software, La Jolla, CA). Statistical analyses conducted between different groups used Student’s t-test assuming two-tailed distributions, with an alpha level of 0.001–0.05. *P < 0.05, **P < 0.01, ***P < 0.001.

## Results

### RAGE knockdown suppresses LPA induced downstream pathways in lung cancer cells

**Fig. 1:**
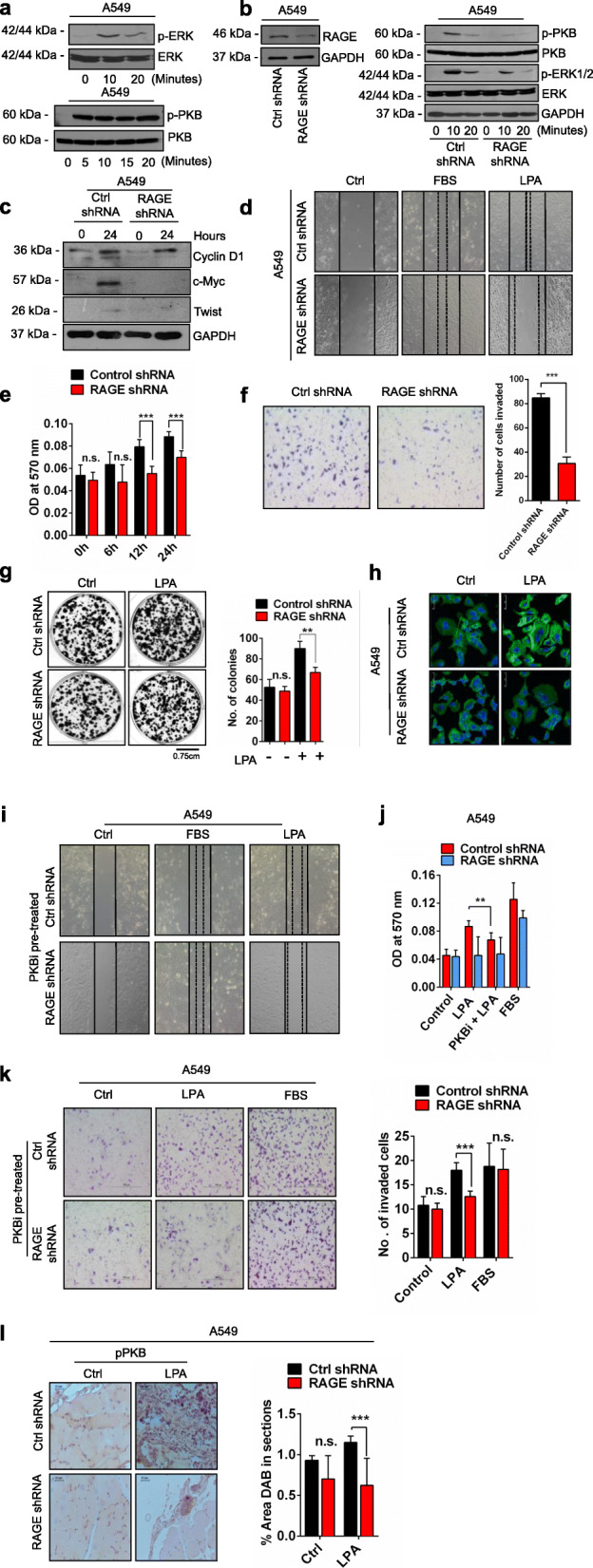
RAGE LPA-RAGE axis mediates cellular proliferation, migration and invasion via PKB signaling pathway in lung cancer cells. **a** Levels of p-ERK/ERK, and p-PKB (Ser473)/PKB, in A549 cells treated with LPA for the indicated time points. **b** Levels of p-ERK/ERK, p-PKB (Ser473)/PKB and GAPDH in A549 cells treated with LPA (5 μM) for the indicated time points after shRNA mediated RAGE silencing. **c** Immunoblots showing levels of cyclinD1, c-myc, twist and GAPDH in A549 cells treated with LPA for the respective time points in hours. **d** Wound healing assay showing the migration of A549 cells stimulated with LPA in untreated, control shRNA and RAGE shRNA transfected cells. **e** Quantitative representation of cell proliferation via MTT assay comparing OD at 570 nm in control shRNA and RAGE shRNA transfected A549 cells in presence and absence of LPA (5 μM) for indicated time points. **f** Transwell invasion of A549 cells after RAGE silencing upon LPA treatment and its quantification. **g** Representative images and quantification of colonies of A549 cells transfected with control shRNA and RAGE shRNA and then grown in medium with or without LPA for 10 days. **h** F-actin polymerization in control and RAGE shRNA A549 cells cultured in medium with or without LPA for 6 h (green, F-actin; blue, DAPI). **i** Wound healing assay showing the migration of RAGE silenced A549 cells pre-treated with PKB inhibitor (LY294002) followed by no treatment (control), LPA and 5% FBS treatment. **j** Quantification of cell proliferation assay of control and RAGE silenced A549 with LPA post PKB inhibitor treatment (LY294002). **k** Trans-well invasion of RAGE silenced A549 cells pre-treated with PKB inhibitor (LY294002) followed by no treatment (control), LPA and 5% FBS treatment with their quantitative representations. **l** Immunohistochemical analysis and quantifications of % area DAB in sections of p-PKB (Ser473) in tumor sections of control shRNA and RAGE shRNA A549 cells intraperitoneally injected nude mice with exogenous LPA treatment. For all experiments, data are means ± SD, *n* = 3–7. **P ≤ 0.01, ***P ≤ 0.001

LPA is a multipotent lipid molecule known to stimulate numerous cellular processes [15, 23, 26]. To study the effect of LPA on lung cancer cells, we stimulated A549 cells with LPA for different time points and found activation of p-ERK and p-PKB with LPA stimulation (Fig. 1a). RAGE is a well-known receptor for LPA and to find out its possible role in lung tumor development, we silenced RAGE in A549 cells using RAGE shRNA with scramble shRNA treated cells as control (Fig. 1b). Further, the treatment of RAGE silenced A549 cells with LPA for different time points showed reduction in phosphorylation of ERK and PKB compared to control shRNA transfected A549 cells (Fig. 1b). Our results confirmed the activation of ERK and PKB pathways in non-small cell lung carcinoma (NSCLC) A549 cells via RAGE.

### LPA induces in vitro lung tumorigenicity via RAGE receptor

Oncogenes are responsible for unregulated cell division and cellular proliferation to establish tumor progression. We investigated the levels of Cyclin D1 and c-Myc oncogenes in A549 cells with LPA treatment. Immunoblot studies of A549 control and RAGE silenced cells with LPA treatments showed that, LPA addition leads to over expression of oncogenes Cyclin D1 and c-Myc via RAGE (Fig. 1c). Further, we also checked the epithelial to mesenchymal transition marker and found twist1 to be upregulated by LPA treatment in A549 cells and RAGE silencing led to decrease in the level of twist1 after LPA treatment suggesting its role in metastasis of A549 cells (Fig. 1c).

Further, to understand the effect of LPA on migration of A549 cells mediated by RAGE, we performed wound healing assay in control and RAGE shRNA treated A549 cells and found that after RAGE silencing LPA stimulation majorly reduced migration of A549 cells compared to control shRNA treated cells (Fig. 1d). These results confirmed that RAGE mediates LPA induced migration of the lung cancer cells.

Cellular proliferation, invasion and clonogenicity are essential indicators of tumor cells ability to disseminate and form secondary metastases. We treated RAGE silenced A549 cells with LPA and checked the proliferation capacity at different time points. We found a significant decrease in cell proliferation after RAGE knockdown at 12 and 24 h of LPA treatment (Fig. 1e). ShRNA treatment had no significant effect on proliferation of A549 cells (Supplementary Fig. 1a). Further, our invasion experiments showed LPA stimulation led to greater invasion of control shRNA treated A549 cells in comparison to RAGE silenced cells (Fig. 1f). These studies confirmed LPA stimulated migration and invasion of A549 cells is mediated by RAGE (Fig. 1f).

Next, to explore the role of LPA and its receptor RAGE in lung cancer cells clonogenic potential, we performed clonogenic assay and grew single cells of control and RAGE silenced A549 cells in medium with and without LPA for 10 days. Our end point analysis revealed greater colony formation of A549 cells treated with LPA compared to control A549 cells and RAGE silenced A549 cells showed reduced number of colonies with LPA treatment (Fig. 1g). These results signify the proliferation capacity of LPA stimulated lung cancer cells to be mediated by RAGE.

### LPA regulates cellular motility of lung cancer cells via RAGE

Actin and microtubules provide active cellular framework to organize and eventually control cellular activation [37]. Actin polymerisation is a measure of the dynamic status of a cell and its readiness for cell division which further leads to higher cell proliferation and tumor metastasis. Therefore, we studied actin polymerization in response to LPA stimulation in A549 cells. LPA addition led to higher actin polymerisation seen by increased fluorescent actin filaments in A549 cells (Fig. 1h). Further, RAGE silenced A549 cells treated with LPA showed reduction in actin polymerisation signifying role of LPA-RAGE in actin polymerisation in lung cells confirming LPA-RAGE axis involvement (Fig. 1h).

### LPA-RAGE axis mediates cellular proliferation, migration and invasion via PKB pathway in lung cancer cells

Next, to identify if the PKB pathway is involved in downstream signaling of LPA-RAGE axis in migration and invasion of A549 cells, we pre-treated control and RAGE knockdown A549 cells with PKB inhibitor and studied migration and invasion on LPA addition. Our results showed reduced migration and proliferation of A549 cells pre-treated with PKB inhibitor followed by LPA treatment. Further RAGE silenced A549 cells pretreated with PKB inhibitor also showed reduced migration and proliferation confirming the role of downstream PKB pathway in LPA-RAGE mediated lung cancer cells migration (Fig. 1i, j). Further, invasion studies using PKB inhibitor in control and RAGE shRNA treated cells stimulated further with LPA also confirmed involvement of PKB pathway via LPA-RAGE axis in A549 cells (Fig. 1k).

In addition, we evaluated p-PKB levels by immunohistochemical analysis in tumor sections of nude mice injected intraperitoneally with control and RAGE knockdown A549 cells and treated further with LPA. We found significantly increased level of p-PKB in LPA treated control A549 tumors compared to RAGE silenced A549 cells tumors in nude mice in vivo (Fig. 1l).

### LPA induces in vivo lung tumorigenicity via RAGE

**Fig. 2:**
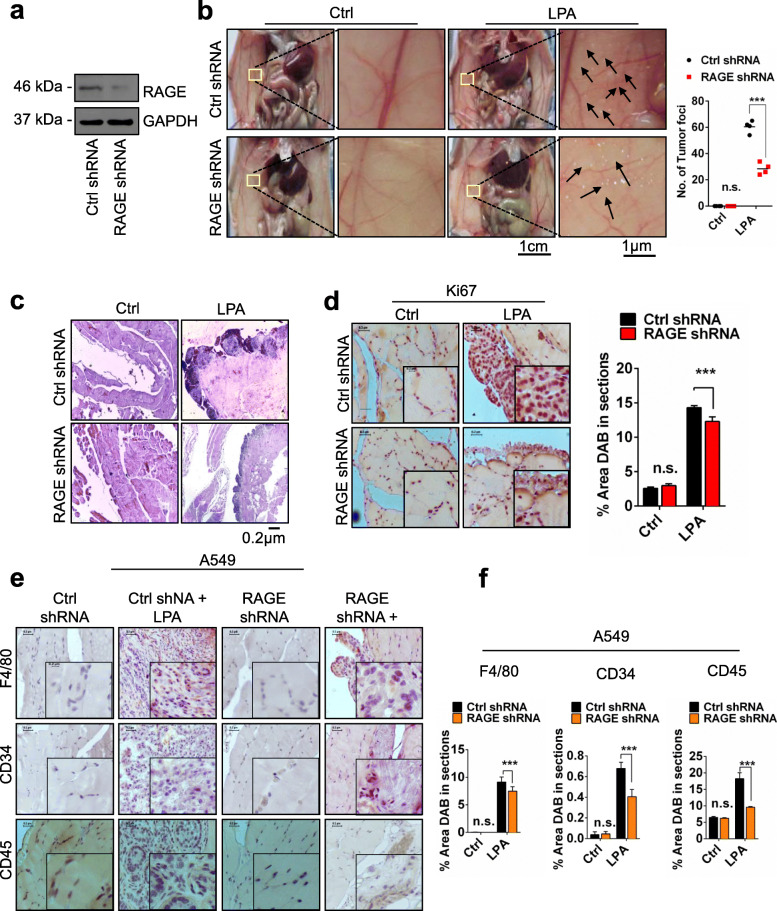
LPA induces in vivo tumorigenicity of lung cancer cells via RAGE. **a** Levels of RAGE in control shRNA and RAGE shRNA treated A549 cells. **b** Left, Representative images showing tumor foci on peritoneal wall of nude mice injected with control shRNA and RAGE treated A549 cells with and without LPA stimulation. Right, Quantification of number of tumor foci on peritoneal wall of nude mice injected with control shRNA and RAGE treated A549 cells with and without LPA stimulation (*n* = 4 for each group). **c** Representative H & E images of tumor section from peritoneal wall of nude mice injected with control shRNA and RAGE treated A549 cells stimulated with or without LPA stimulation. **d** Representative images show Ki67 immunohistochemical staining of the tumor sections of nude mice injected with control shRNA and RAGE treated A549 cells with and without LPA stimulation and its quantitative representation. **e**, **f** Representative images show markers for macrophage (F4/80), angiogenesis (CD34) and leukocyte (CD45) infiltration in control shRNA and RAGE shRNA A549 tumors and quantification of % area DAB in sections of mice treated with LPA and their quantifications. For all experiments, data are means ± SD., ***P ≤ 0.001

To find out if RAGE is involved in LPA induced lung cancer progression in vivo and further to investigate the physiological significance of LPA-RAGE signaling in lung tumor formation in vivo, we injected scrambled (control) and RAGE silenced A549 cells into the peritoneal cavity of nude mice (Fig. 2a and b). These mice were injected with LPA every alternate day for the next 21 days. End point examination of the peritoneal wall and cavity of nude mice injected with scrambled A549 cells showed increased tumor formation with significantly higher tumor numbers and size compared to RAGE knockdown A549 cells injected mice after LPA treatment (Fig. 2b). The haematoxylin and eosin staining of the paraffin embedded sections of peritoneal wall showed higher numbers of fragmented nuclei in the sections of nude mice injected with control shRNA after LPA stimulation than RAGE shRNA cells. PBS treated control showed lesser fragmented nuclei in the section than the LPA treated, establishing LPA act as lung tumor promoter (Fig. 2c). Further, higher levels of proliferation marker Ki67 were observed in LPA treated control shRNA treated A549 injected nude mice than the RAGE shRNA A549 injected LPA treated nude mice (Fig. 2d).

In addition, immunohistochemical analysis of the tumors in nude mice injected with control and RAGE shRNA treated A549 cells showed significantly high angiogenesis (CD34), leukocyte (CD45) and macrophage (F4/80) levels in control shRNA compared to RAGE shRNA A549 injected nude mice and treated further with LPA (Fig. 2e and f) confirming role of RAGE in LPA mediated lung tumorigenesis and immune cells infiltration in tumor microenvironment.

### LPA via RAGE promotes in vitro mammary tumorigenicity

**Fig. 3:**
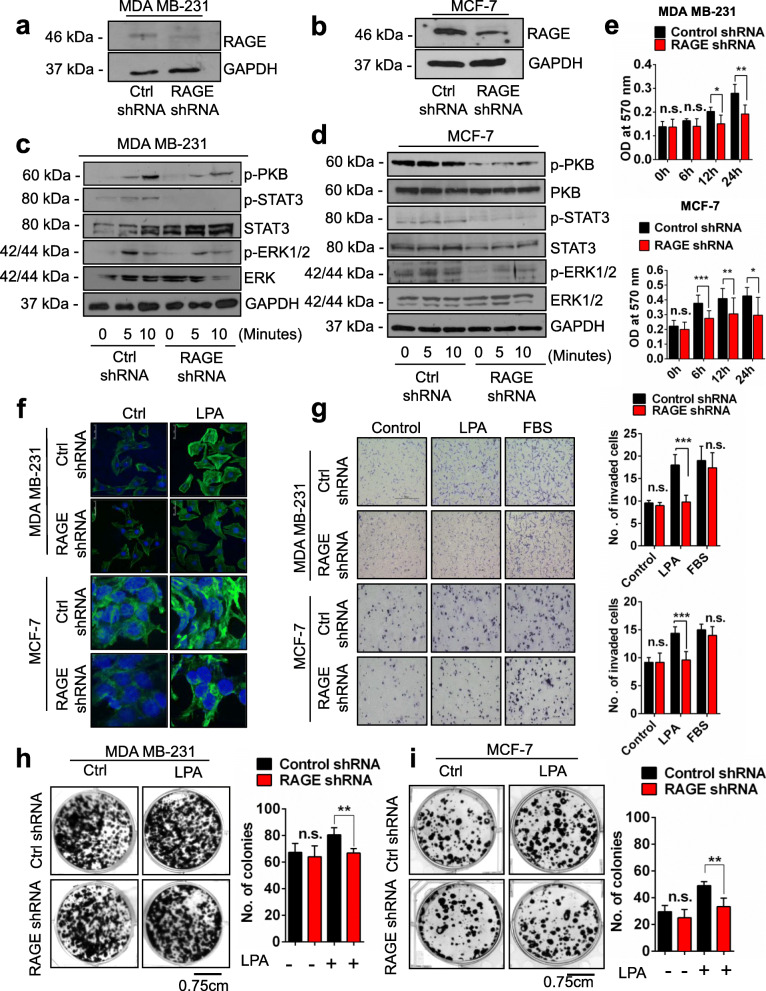
RAGE knockdown suppresses LPA induced downstream pathways in mammary cancer cells. **a**, **b** Levels of RAGE in mammary cancer cells (MDA MB-231 and MCF-7, respectively) after shRNA mediated RAGE silencing. **c**, **d** Levels of p-ERK/ERK, and p-PKB (Ser473)/PKB, p-STAT3/STAT3 and GAPDH in mammary cancer cells (MDA MB-231 and MCF-7, respectively) treated with LPA for the indicated time points after shRNA mediated RAGE silencing. **e** Quantification of cell proliferation assay of control and RAGE silenced A549 with LPA post PKB inhibitor treatment (LY294002). **f** F-actin polymerization in control and RAGE shRNA MDA MB-231 and MCF-7 cells cultured in medium with or without LPA for 6 h (green, F-actin; blue, DAPI). **g** Representative images and quantifications of transwell invasion assays of control and RAGE shRNA treated MDA MB-231 and MCF-7 cells after LPA treatment. **h**, **i** Representative images and quantification of colonies of MDA MB-231 and MCF-7 cells treated with control shRNA and RAGE shRNA and then grown in medium with or without LPA for 10 days. (For all experiments, data are means ± SD., *n* = 3–7. * ≤ 0.05, **P ≤ 0.01, ***P ≤ 0.001)

To extend our studies and delineate LPA-RAGE axis possible role in breast cancer, we tested effect of LPA on metastatic breast cancer MDA MB-231 cells and poorly-aggressive and non-invasive MCF-7 cells with low metastatic properties [38]. We silenced RAGE in MDA MB-231 and MCF-7 cells using RAGE shRNA with scramble shRNA treated cells as control (Fig. 3a & b). Further, the treatment of control and RAGE silenced MDA MB-231 and MCF-7 cells with LPA for different time points showed reduction in phosphorylation of ERK and PKB in RAGE silenced compared to control MDA MB-231 and MCF-7 cells (Fig. 3c & d). These results confirmed the activation of ERK and PKB pathways in MDA MB-231 and MCF-7 cells via RAGE. Furthermore, we also observed activation of STAT3 in control cells treated with LPA in comparison to RAGE silenced MDA MB-231 and MCF-7 cells (Fig. 3c & d).

Next, to determine the role of LPA in proliferation of MDA MB-231 and MCF-7 cells, we treated MDA MB-231 and MCF-7 cells with LPA and found that LPA induced proliferation of these cells in MTT assay colorimetric quantification. RAGE silencing in MDA MB-231 and MCF-7 cells showed reduced proliferation in both of these cells after LPA stimulation suggesting role of RAGE in MDA MB-231 and MCF-7 cells proliferation capacity (Fig. 3e). In addition, we also assessed the effect of shRNA treatment on proliferation of MDA MB-231 and MCF-7 cells and found no significant effect of shRNA treatment on the proliferation of these cells (Supplementary Fig. 1b, c).

In addition, we also studied actin polymerization in response to LPA stimulation in MDA MB-231 and MCF-7 cells. LPA addition led to higher actin polymerisation, seen by increased fluorescent actin filaments in MDA MB-231 and MCF-7 cells (Fig. 3f). RAGE silenced MDA MB-231 and MCF-7 cells treated with LPA showed reduction in actin polymerisation signifying role of LPA-RAGE in actin polymerisation in breast cancer cells (Fig. 3f). Further, we investigated role of LPA in breast cancer cells invasion. LPA stimulation led to greater invasiveness of MDA MB-231 and MCF-7 cells while RAGE silencing in these cells reduced their invasive properties confirming RAGE mediated LPA induced invasion (Fig. 3g). Next, to examine the effect of LPA on clonogenic properties of mammary tumor cells, control and RAGE knockdown cells were grown in medium in absence or presence of LPA. Our end point analysis revealed high colony formation ability of MDA MB-231 and MCF-7 cells treated with LPA compared to control. Further, RAGE silenced MDA MB-231 and MCF-7 cells showed lesser number of colonies signifying RAGE mediated effect of LPA on clonogenic potential (Fig. 3h & i).

### PKB pathway is involved in LPA-RAGE mediated migration, proliferation and invasion of mammary tumor cells

To understand the effect of LPA on migration of MDA MB-231 and MCF-7 cells mediated by RAGE, we performed wound healing assay in control and RAGE silenced cells and found that after RAGE silencing LPA stimulation majorly reduced migration of MDA MB-231 and MCF-7 cells compared to control shRNA treated cells (Supplementary Fig. 2a).

**Fig. 4:**
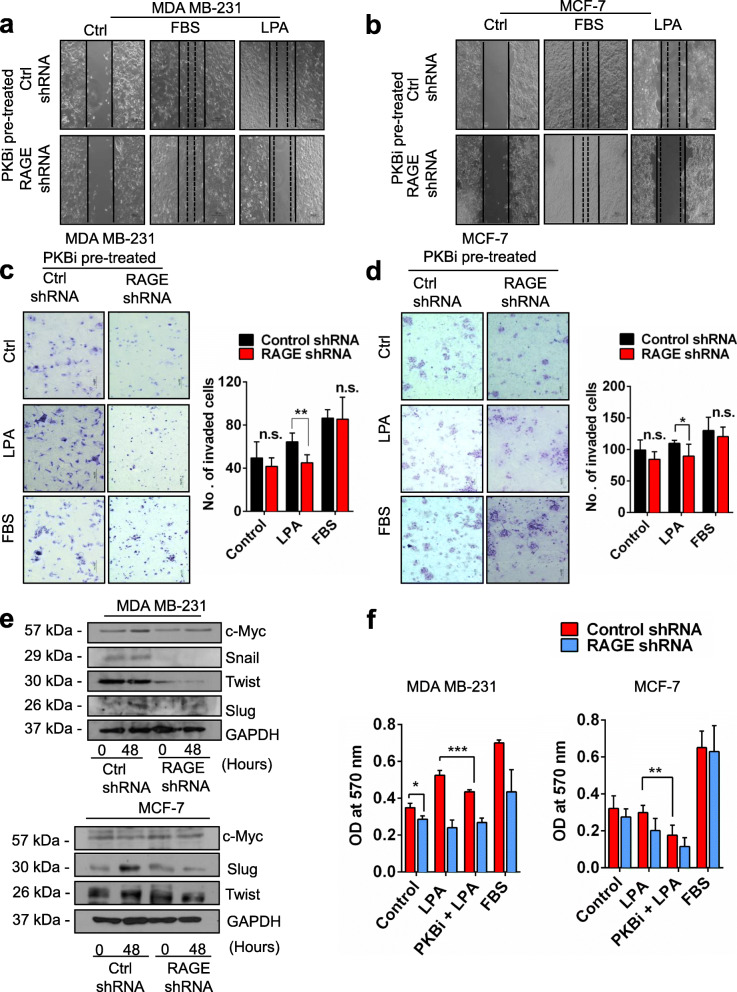
LPA-RAGE axis mediates cellular proliferation, migration and invasion via PKB signaling pathway in mammary cancer cells. **a**, **b** Wound healing assay showing the migration of RAGE silenced MDA MB-231 and MCF-7 cells pre-treated with PKB inhibitor (LY294002) followed by no treatment (control), LPA and 5% FBS treatment. **c**, **d** Trans-well invasion of RAGE silenced MDA MB-231 and MCF-7 cells pre-treated with PKB inhibitor (LY294002) followed by no treatment (control), LPA and 5% FBS treatment with their quantitative representations. **e** Immunoblots showing the c-Myc, Slug, Twist, and GAPDH expression in control shRNA and RAGE shRNA treated MDA MB-231 and MCF-7 cells with and without LPA stimulation. **f** Quantification of cell proliferation assay of control and RAGE silenced MDA MB-231 and MCF-7 with LPA post PKB inhibitor treatment (LY294002). For all experiments, data are means ± SD., n = 3–7. * ≤ 0.05, **P ≤ 0.01, ***P ≤ 0.001

Next, to validate if the PKB pathway as observed in lung cancer cells is also involved in downstream signaling of LPA-RAGE axis in migration and invasion of MDA MB-231 and MCF-7 cells, we pre-treated control and RAGE knockdown MDA MB-231 and MCF-7 cells with PKB inhibitor and studied their migration and invasion on LPA addition. Our results showed reduced migration of MDA MB-231 and MCF-7 cells pre-treated with PKB inhibitor and LPA (Fig. 4a & b). RAGE silenced cells pretreated with PKB inhibitor showed reduced migration (Fig. 4a & b). Further, invasion studies using PKB inhibitor in control and RAGE shRNA treated cells stimulated further with LPA showed involvement of PKB pathway via LPA-RAGE axis in these cells (Fig. 4c & d). These studies confirmed the role of downstream PKB pathway in LPA-RAGE mediated mammary cancer cells migration and invasion.

Next, to investigate the effect of LPA on oncogene via RAGE, we checked the levels of c-Myc in control and RAGE silenced MDA MB-231 and MCF-7 cells after LPA treatment. Immunoblot studies with LPA treated control and RAGE silenced cells showed that LPA addition leads to upregulation of c-Myc via RAGE (Fig. 4e).

Furthermore, to confirm if PKB pathway is involved downstream of LPA-RAGE for MDA MB-231 and MCF-7 cells proliferation, we pre-treated these cells with PKB inhibitor and observed for proliferation on LPA addition. Our results showed reduced proliferation of MDA MB-231 and MCF-7 cells pre-treated with PKB inhibitor and LPA in comparison with no PKB inhibitor treatment, confirming the role of downstream PKB pathway in LPA-RAGE mediated mammary tumor cancer cells proliferation (Fig. 4f).

### LPA regulates epithelial-mesenchymal transition in mammary cancer cells via RAGE

Epithelial to mesenchymal transition markers have been found to be associated with cancer progression and metastasis [21]. Immunohistochemical analysis of EMT markers E-cadherin and Vimentin in the tumor sections of nude mice receiving intraperitoneal injection of control and RAGE shRNA treated A549, MDA MB-231 and MCF-7 cells and treated with LPA revealed higher expression of epithelial maker E-cadherin in untreated ones than the LPA treated nude mice. The mesenchymal marker Vimentin expression was significantly higher in tumor sections of nude mice receiving control shRNA treated cells followed by LPA treatments than the RAGE silenced cells injected nude mice tumor sections confirming epithelial to mesenchymal transition through RAGE (Supplementary Fig. 2b). Further, EMT promoting transcription factors, Snail, Slug and Twist1 showed induced expression upon LPA treatment as shown in immunoblots (Fig. 4e). In MDA MB-231 cells we found induction of Snail and Slug with LPA stimulation which was significantly reduced in RAGE silenced cells (Fig. 4e). Further, LPA induction in MCF-7 cells upregulated expression of slug and twist which was significantly reduced on RAGE silencing (Fig. 4e).

### LPA via RAGE promotes in vivo mammary tumorigenicity

**Fig. 5:**
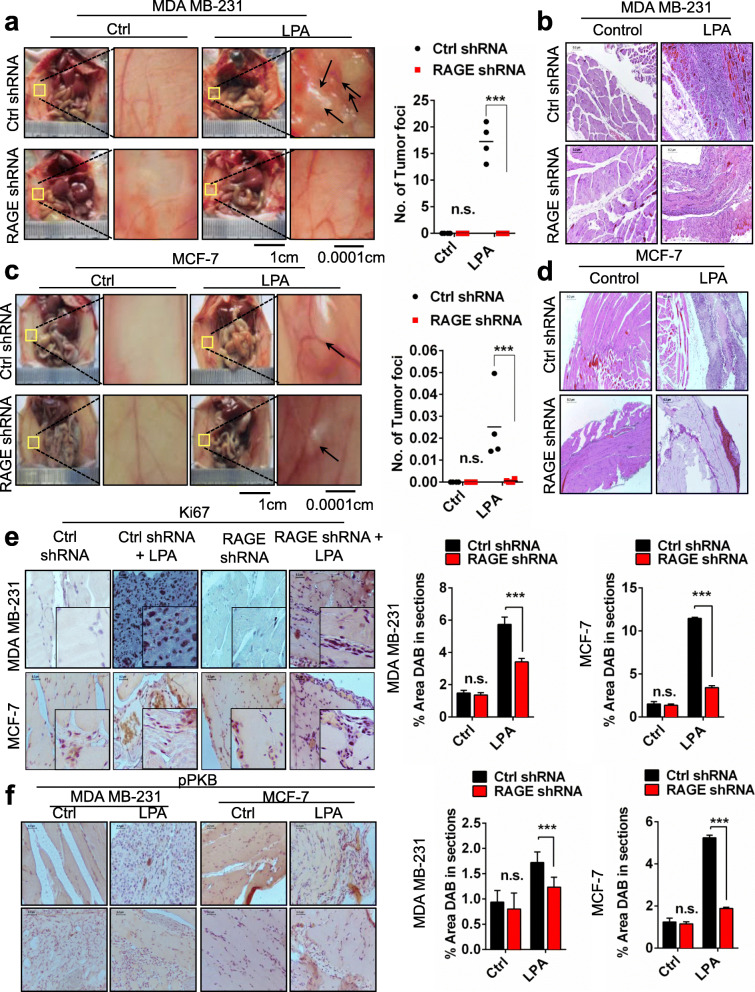
LPA induces in vivo mammary tumorigenicity via RAGE. **a**, **b**, **c**, **d** Representative images showing tumor foci on peritoneal wall of nude mice injected with control shRNA and RAGE treated MDA MB-231 and MCF-7 cells with and without LPA stimulation. Right, Quantification of number of tumor foci on peritoneal wall of nude mice injected with control shRNA and RAGE treated MDA MB-231 and MCF-7 cells with and without LPA stimulation (n = 4 for each group). **e** Representative images show Ki67 immunohistochemical staining of the tumor sections of nude mice injected with control shRNA and RAGE treated MDA MB-231 and MCF-7 cells with and without LPA stimulation and its quantitative representation. **f** Immunohistochemical analysis and quantifications of % area DAB in sections of p-PKB (Ser473) in tumor sections of control shRNA and RAGE shRNA MDA MB-231 and MCF-7 cells intraperitoneally injected nude mice with exogenous LPA treatment. For all experiments, data are means ± SD., *n* = 4. ***P ≤ 0.001

Further to test the effect of LPA via RAGE in mammary tumors in vivo, we implanted scrambled (control) and RAGE silenced MDA MB-231 and MCF-7 cells into the peritoneum of nude mice and administered exogenous LPA through intraperitoneal injections for 21 days. End point analysis revealed increased tumor formation with LPA treatment in nude mice transplanted with control shRNA treated MDA MB-231 (Fig. 5a & b) and MCF-7 cells (Fig. 5c & d) compared to RAGE shRNA treated cells confirming tumor formation via RAGE.

The immunohistochemical staining of the peritoneal wall tumor sections with Ki67 antibody and its quantitative analysis showed significantly higher levels of Ki67 in tumor sections of nude mice transplanted with control shRNA treated MDA MB-231 and MCF-7 receiving LPA treatments than the RAGE shRNA treated MDA MB-231 and MCF-7 cells (Fig. 5e).

Further, we evaluated the p-PKB levels by immunohistochemical analysis in tumor sections of these nude mice and found significantly increased level of p-PKB in LPA treated control MDA MB-231 and MCF-7 cells tumors compared to RAGE silenced MDA MB-231 and MCF-7 cells tumors in vivo (Fig. 5f).

### Metastasis and immune cells infiltration in a xenograft model of mammary cancer cells

**Fig. 6:**
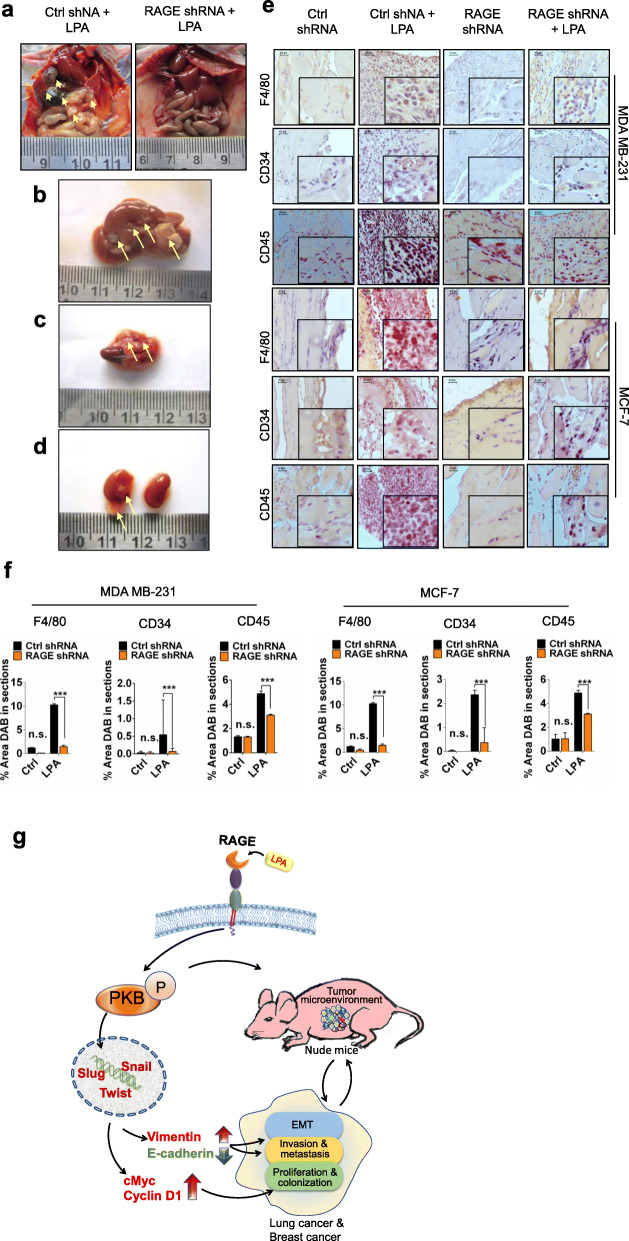
Metastasis and immune cells infiltration in a xenograft model of mammary cancer cells. **a** Representative images show metastasis (Yellow arrows) of MDA MB-231 cells in peritoneal cavity of nude mice injected with control and RAGE shRNA treated MDA MB-231 cells after exogenous LPA treatments. Representative images of (**b**) liver, (**c**) lungs with heart and (**d**) kidneys of nude mice show metastasis of control shRNA treated MDA MB-231 upon LPA induction. **e**, **f** Representative images show markers for macrophage (F4/80), angiogenesis (CD34) and leukocyte (CD45) infiltration in control shRNA and RAGE shRNA MDA MB-231 and MCF-7 tumors and quantification of % area DAB in sections of mice treated with LPA and their quantifications. **g** Schematic representation of LPA via RAGE promotes lung and mammary carcinogenesis and epithelial to mesenchymal transition trough the PKB pathway. For all experiments, data are means ± SD., n = 3–7. ***P ≤ 0.001

Further to check metastasis in our in vivo experiments, we examined the possible dissemination of tumor cells to other tissues. Our results showed the metastasis of MDA MB-231 cells from peritoneal cavity to other visceral organs. The MDA MB-231 metastatic tumors were observed after LPA treatment on the visceral organs viz. lung, liver duodenum and kidney of the nude mice injected with control shRNA treated cells while the RAGE shRNA treated cells did not show any metastasis on LPA stimulation (Fig. 6a, b, c & d). Immunohistochemical analysis of the tumors in control and RAGE shRNA treated cells injected in nude mice showed significantly higher angiogenesis (CD34), leukocyte (CD45) and macrophage (F4/80) markers level in control shRNA compared to RAGE shRNA treated MDA MB-231 and MCF-7 (Fig. 6e, f) injected nude mice and treated further with LPA confirming role of RAGE in LPA mediated mammary tumorigenesis and immune cells infiltration in tumor microenvironment.

## Discussion

RAGE is a pattern recognition receptor involved in non-small cell lung cancer tumorigenesis and its metastasis. LPA is a recently discovered ligand of RAGE and is involved in several physiological and pathophysiological conditions [15]. RAGE and its ligands interact and activate downstream signaling pathways such as JAK/STAT, MAPK and NF-κB in various cancers [21]. However, studies are lacking on its recently identified ligand LPA via RAGE axis in lung and breast cancer tumorigenesis. Based on comprehensive in vitro and in vivo studies we identify previously unknown LPA-RAGE axis and we show that, in non-small cell lung carcinomas (NSCLC) or A549 cells, LPA activates PKB and ERK pathways via RAGE. LPA induces migration, invasion and proliferation of NSCLC cells via RAGE and downstream PKB pathways which have been confirmed by silencing and inhibition of RAGE and PKB. Our study shows that LPA induces NSCLC cell tumors and regulates its microenvironment and EMT via RAGE in vivo using xenograft model. Our study confirms involvement of LPA via its receptor RAGE in the progression and migration of lung cancer cells tumor. Further, it confirms the downstream involvement of PKB pathway in the LPA-RAGE signaling. Additionally, we have explored the LPA-RAGE axis further in tumorigenesis of breast cancer cells. We have identified that similar signaling pathway i.e., PKB mediated migration, invasion and proliferation of breast cancer cells in vitro. Our LPA induced in vivo results in breast cancer cells show involvement of RAGE mediated mechanisms as lung cancer cell tumorigenesis.

It has been shown that RAGE promotes tumor growth and metastasis of NSCLC cells by regulating β-catenin signaling pathways [39]. In case of breast cancer, RAGE-ligand interaction in MDA MB-231 and MCF7 cells promotes ERK1/2 signaling and NF-κB signaling and increased cell migration and invasion [40]. Studies have shown the role of LPA and its receptors in tumor progression and metastasis in various cancers; whereas LPA-RAGE signaling in lung and breast cancer development and modulation of tumor microenvironment had not been explored [41]. This study identifies, LPA induced PKB and ERK signaling pathways involve in lung adenocarcinoma cells (A549) and further, RAGE silencing confirms the LPA-RAGE axis in regulation of downstream signaling in A549 cells. Furthermore, LPA drives upregulation of EMT markers (vimentin, and twist), oncogene (c-Myc) and downregulation of epithelial marker E-cadherin. In MDA MB-231 and MCF7 cells, LPA upregulates EMT markers (vimentin, slug, snail and twist), oncogene (c-Myc) and decrease in E-cadherin via RAGE. EMT transition involves various cellular properties such as migration, proliferation, tissue invasion which promote the cancerous cells to grow, proliferate and spread to different organs/sites [20, 42]. Our findings show that LPA induces cellular migratory and invasive properties of lung and breast cancer cells via RAGE in vitro. Actin polymerisation is a measure of the dynamic status of a cell, epithelial mesenchymal transition and its readiness for cell division which further leads to higher cell proliferation and tumor metastasis [37, 43]. To decipher the role of LPA in inducing these cellular properties are further validated by higher actin polymerization and PKB inhibition suggests LPA-RAGE axis induces in vitro tumorigenic properties of tumor cells are PKB-dependent.

RAGE silenced in the highly metastatic MDA-MB-231-4175 cells decreased tumor growth at the orthotopic site and prevented metastasis of MDA MB-231 and MDA MB-231-4175 [20]. Furthermore, tail vein injection of S100a8/a9, together with MDA MB-231 cells showed the enhanced invasion/ metastases [44]. However, no studies to date have explored the role of LPA-RAGE axis in in vivo tumor development by lung and breast cancer cells. Here, we investigated the impact of LPA-RAGE axis in tumor development in xenograft nude mice injected with A549, MDA MB-231 and MCF-7 cells. Our study showed that mice treated with LPA have higher number and size of tumors on peritoneum wall of mice. Moreover, RAGE silencing in injected cancer cells shows no or small tumor foci on the surface of peritoneum wall. Our results confirmed the PKB pathway involvement in LPA mediated lung and mammary tumor progression via RAGE.

Understanding how LPA-RAGE signaling regulates the two-way communication between tumor cells and their microenvironment that promote tumor development is important as it can be an attractive therapeutic approach to target different mechanisms that promote tumor progression. In this study, our data show that LPA treatment induce angiogenesis, proliferation and mobilization of inflammatory cells to the tumor; however, RAGE knockdown of lung and breast cancer cells leads to reduction of these markers in tumor tissues. Earlier studies have demonstrated importance of RAGE and its ligands in regulation of tumor microenvironment and inflammation and RAGE silencing in skin carcinoma and colitis-associated cancer reduce recruitment of inflammatory cell and tumor progression [45, 46]. Earlier, Nassar et al. have shown RAGE stimulated with its ligand S100A7 promote the breast cancer progression and metastasis by transforming its tumor microenvironment [47]. It needs to be noted that our results do not rule out possible involvement of other LPA receptors or any component associated with the classical receptors as our results do not show complete reduction in cellular and tumorigenic properties on RAGE silencing, but RAGE appears to be a major player in these cancers. Further recent reports also show RAGE transactivation following activation of the AT1R, a classical G proteins-coupled receptor which can be an area of potential interest in future studies in these cancers [48, 49]. However, for the first time, this study has revealed the role LPA-RAGE signaling in lung and breast cancer cells in regulation of tumor microenvironment.

## Conclusion

Our study identifies previously unrecognised LPA-RAGE axis and its signaling pathway involved in tumorigeneis and metastasis in lung and mammary cancer cells. Our study shows that, LPA-RAGE signaling is a key promoter of intrinsic cellular properties of lung and mammary cancer cells such as proliferation, migration, tissue invasion and metastasis. Furthermore, this study provides critical support that PKB pathway affects tumor cell invasion, proliferation, migration, and colonization and also by regulating tumor microenviroment in these tumors. However, further studies are required to identify the role of LPA-RAGE signaling in stromal communication involve in lung and breast cancer inflammation. Taken together, present findings provide evidence that LPA-RAGE axis could be future therapeutic target for controlling the progression and metastasis of lung and breast cancer (Fig. 6g).

## Supplementary information

**Additional file 1: Fig. S1.** Proliferation of lung and breast cancer cells after RAGE inhibition. Quantitative representation of cell proliferation via MTT assay comparing OD at 570 nm in untreated cells and control shRNA treated (a) A549, (b) MDA-MB-231 and (c) MCF-7 cells without any stimulation and with LPA treatment after 24 h. For all experiments, data are means ± SD., n.s., not significant.**Fig. S2.** LPA-RAGE axis mediates lung and mammary tumor microenvironment. (a) Wound healing assay showing the migration of MDAMB-231 and MCF-7 cells stimulated with LPA in untreated, control shRNA and RAGE shRNA treated cells. (b) Representative images and quantification of percentage area DAB in sections of immunohistochemical staining of E-cadherin and Vimentin in tumor sections of nude mice injected with control shRNA and RAGE shRNA treated A549, MDA MB 231 and MCF-7 cells with and without LPA stimulation. *n* = 5 for each group. For all experiments, data are means ± SD., *n* = 3–7. ***P ≤ 0.001.

## Data Availability

All data generated or analysed during this study are included in this article and supplementary informations.

## Abbreviations

AGEs: Advanced glycation end products
PKB: Protein kinase B
DAB: 3,3′-diaminobenzidine
EMT: Epithelial Mesenchymal Transition
ERK1/2: Extracellular-signal-regulated kinase
GPCRs: G-protein coupled receptors
IHC: Immunohistochemistry
ICC: Immunocytochemistry
LPA: Lysophosphatidic Acid
p38: MAP kinase
JAK: Janus Kinase
NSCLC: Non-small cell lung carcinoma
MAPK: Mitogen-activated protein kinases
mDia 1: Mammalian diaphanous 1
MTT: 3-(4,5-Dimethylthiazol-2-Yl)-2,5-Diphenyltetrazolium Bromide
NFκB: Nuclear factor κB
Rac: Rac GTPase
RAGE: Receptor for advanced glycation end products
Ras: Rat Sarcoma GTPase
S100: S100 proteins
STAT3: Signal transducer and activation of transcription 3
SAPK: Stress activated protein kinase
TRPV1: Transient receptor potential cation channel subfamily V member 1
