# Supplementary material for: Lysophosphatidic acid-RAGE axis promotes lung and mammary oncogenesis via protein kinase B and regulating tumor microenvironment
Source: Cell Commun Signal. 2020 Oct 27;18:170. doi: 10.1186/s12964-020-00666-y (PMC7592382; doi:10.1186/s12964-020-00666-y)
Supplement: Supplementary file 2 — Additional file 1: Fig. S1. Proliferation of lung and breast cancer cells after RAGE inhibition. Quantitative representation of cell proliferation via MTT assay comparing OD at 570 nm in untreated cells and control shRNA treated (a) A549, (b) MDA-MB-231 and (c) MCF-7 cells without any stimulation and with LPA treatment after 24 h. For all experiments, data are means ± SD., n.s., not significant.Fig. S2. LPA-RAGE axis mediates lung and mammary tumor microenvironment. (a) Wound healing assay showing the migration of MDAMB-231 and MCF-7 cells stimulated with LPA in untreated, control shRNA and RAGE shRNA treated cells. (b) Representative images and quantification of percentage area DAB in sections of immunohistochemical staining of E-cadherin and Vimentin in tumor sections of nude mice injected with control shRNA and RAGE shRNA treated A549, MDA MB 231 and MCF-7 cells with and without LPA stimulation. n = 5 for each group. For all experiments, data are means ± SD., n = 3–7. ***P ≤ 0.001. [file 12964_2020_666_MOESM2_ESM.pptx]

## Slide 1
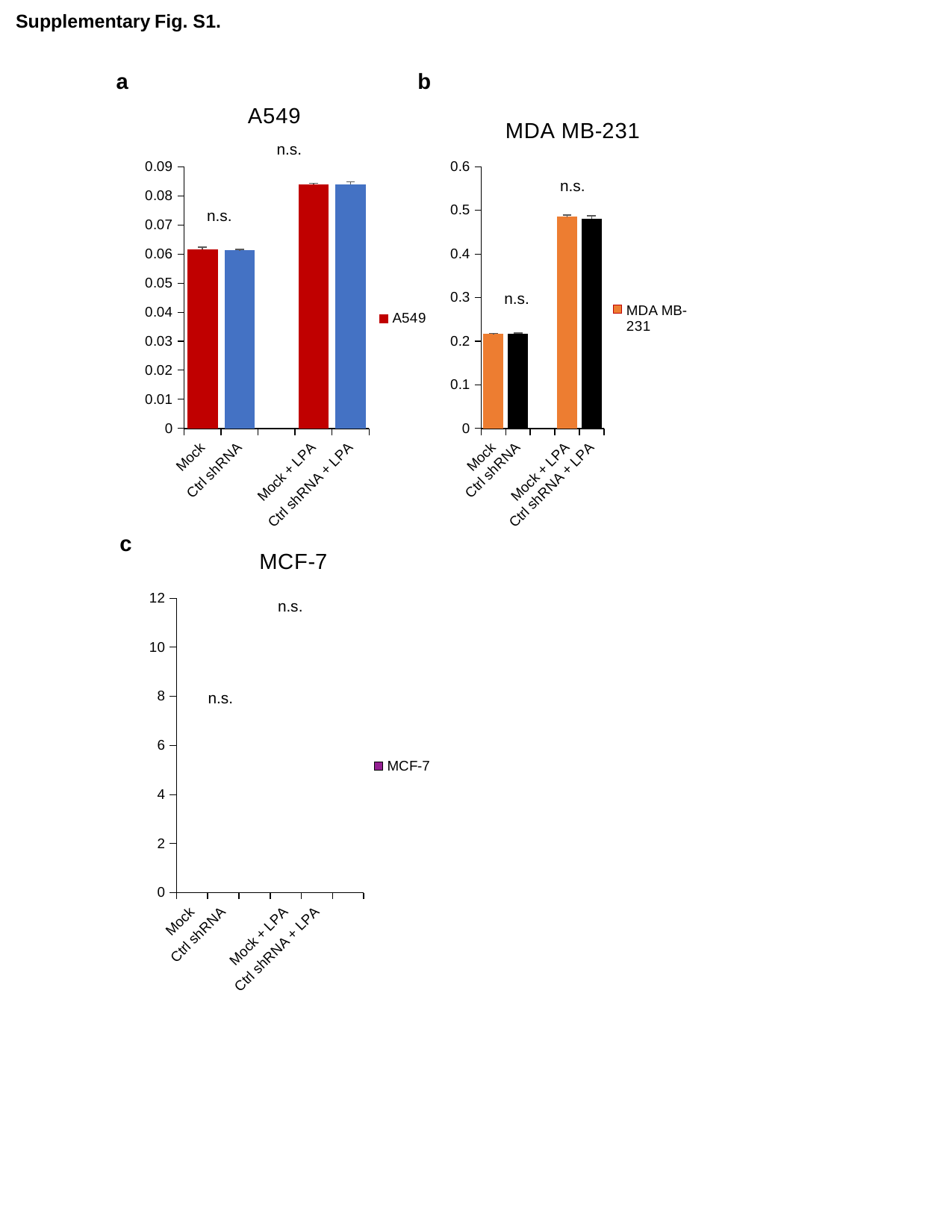

Supplementary Fig. S1.
b
a
### Chart:
| Category | A549 |
|---|---|
| Mock | 0.06143696 |
| Ctrl shRNA | 0.06114000000000001 |
| | None |
| Mock + LPA | 0.08375999999999999 |
| Ctrl shRNA + LPA | 0.08384 |
### Chart:
| Category | MDA MB-231 |
|---|---|
| Mock | 0.21556161999999998 |
| Ctrl shRNA | 0.21680000000000002 |
| | None |
| Mock + LPA | 0.48419999999999996 |
| Ctrl shRNA + LPA | 0.48040000000000005 |n.s.
n.s.
n.s.
n.s.
c
### Chart:
| Category | MCF-7 |
|---|---|
| Mock | 0.16480820000000002 |
| Ctrl shRNA | 0.162 |
| | None |
| Mock + LPA | 0.2568 |
| Ctrl shRNA + LPA | 0.25439999999999996 |n.s.
n.s.

## Slide 2
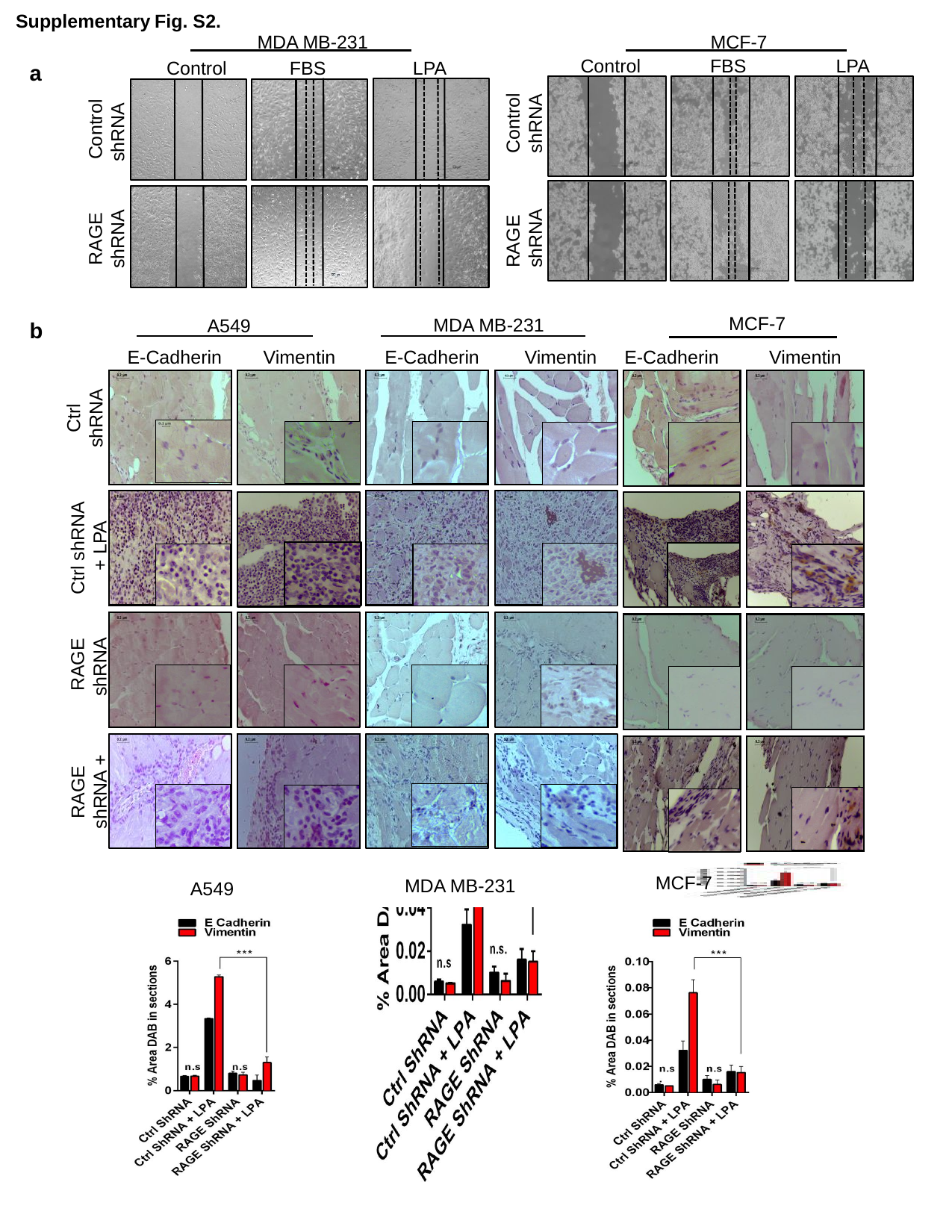

Supplementary Fig. S2.
MDA MB-231
Control
 FBS
LPA
 Control shRNA
RAGE shRNA
MCF-7
Control
 FBS
LPA
Control shRNA
RAGE shRNA
a
MCF-7
E-Cadherin
Vimentin
MDA MB-231
A549
E-Cadherin
Vimentin
E-Cadherin
Vimentin
Ctrl shRNA
Ctrl shRNA
 + LPA
RAGE shRNA
RAGE shRNA + LPA
b
MCF-7
MDA MB-231
A549
